# Correlates of Gambling Behaviour Among Adolescents: The Role of Psychological Factors, School Behaviours, and Normative Perceptions

**DOI:** 10.3390/bs15050653

**Published:** 2025-05-12

**Authors:** Mariaelisa Renna, Emina Mehanović, Giulia Giraudi, Alberto Sciutto, Erica Viola, Marco Martorana, Serena Vadrucci, Maria Ginechesi, Claudia Vullo, Adalgisa Ceccano, Chiara Andrà, Pietro Casella, Fabrizio Faggiano, Federica Vigna-Taglianti, the GAPUnplugged Coordination Group

**Affiliations:** 1 Department of Translational Medicine, University of Piemonte Orientale, 28100 Novara, Italy; 20042790@studenti.uniupo.it (M.R.); federica.vignataglianti@uniupo.it (F.V.-T.); 2 Department of Sustainable Development and Ecological Transition, University of Piemonte Orientale, 13100 Vercelli, Italy; 3 Department of Statistics, Computer Science, and Applications “Giuseppe Parenti” (DiSIA), University of Florence, 50134 Firenze, Italy; 4 Hygiene and Public Health Unit, Department of Prevention, ASL Città di Torino, 10128 Torino, Italy; 5 Addiction Unit, Department of Mental Health, ASL Roma1, 00161 Roma, Italy; 6 Epidemiology Unit, ASL Vercelli, 13100 Vercelli, Italy

**Keywords:** adolescents, gambling, psychological factors, school behaviours, norms

## Abstract

Background: Gambling risk behaviour is an emerging problem among adolescents. This study investigated the role of psychological factors, school behaviours, and normative perceptions as correlates of gambling among 12–14-year-old students in Italy. Methods: The study included 1822 students from 29 secondary schools in two Italian Regions (Piedmont and Lazio) who participated in the baseline survey of the experimental controlled trial “GAPUnplugged”. Results: The prevalence of gambling in the last 30 days was 36.4%. The mean age was 13.1 years. Multilevel mixed-effect regression models identified high positive attitudes, high performance beliefs, low risk perceptions toward gambling, friends’ gambling, friends’ approval of gambling, and gambling with friends as independent correlates of adolescent gambling behaviour. Conclusions: It appears essential to design and implement preventive strategies addressing these factors among early adolescents in order to reduce gambling behaviours and their consequences in later ages.

## 1. Introduction

Gambling behaviour among adolescents is recognized as a public health problem (Armitage, 2021; Delfabbro et al., 2016). Gambling experiences among adolescents begin at an early age, and early gamblers have a higher risk of gambling-related consequences at later ages (Burge et al., 2006; Rahman et al., 2012). Over the years, gambling has become highly accessible, primarily due to the rise in online forms of gambling, such as the “freemium” video games and online sports betting (Andrie et al., 2019; Observatorio Español de las Drogas y las Adicciones, 2021). Furthermore, loot boxes, i.e., an in-game reward system that can be purchased repeatedly with real money to obtain a random selection of virtual items, have become very common on mobile platforms, contributing to gambling behaviour among adolescents and youth (King & Delfabbro, 2018; Montiel et al., 2022; Rockloff et al., 2021).

Moreover, advertising plays a significant role in promoting the behaviour, as it portrays gambling as socially desirable, entertaining, and a source of “easy money” (McMullan & Miller, 2010; O’Loughlin & Blaszczynski, 2018; Sklar & Derevensky, 2011). As a consequence, adolescents often consider gambling a fun entertainment, a way to not become bored, a recreational activity, and finally, perceive it as risk-free (McGrane et al., 2023; Vegni et al., 2019).

Several factors have been identified as correlates of adolescent gambling. Among socio-demographic characteristics, male gender, being part of an immigrant family, and being the only child were associated with gambling in previous studies (Anagnostopoulos et al., 2017; Dickson et al., 2008; Frisone et al., 2020; Giralt et al., 2018; Pisarska & Ostaszewski, 2020; Sharman et al., 2019). Among psychological factors, attitudes, beliefs, expectancies, and risk perceptions played an important role (Botella-Guijarro et al., 2020; Delfabbro et al., 2006; Delfabbro et al., 2009a; Delfabbro & Thrupp, 2003; Derevensky & Gilbeau, 2015; Donati et al., 2013; Favieri et al., 2023; Griffiths & Wood, 2000; Hanss et al., 2014; Hurt et al., 2008; Pallesen et al., 2016). Moreover, problematic stressful situations, negative life events, depression or anxiety symptoms, negative mood and low self-esteem were also associated with engagement in gambling (Delfabbro et al., 2006; Nigro et al., 2017; Stefanovics et al., 2023), as well as maladaptive coping strategies, poor self-control, impulsiveness, high sensation-seeking and risky activity propensity (Bergevin et al., 2006; Dickson et al., 2008; Dowling et al., 2017; Kaltenegger et al., 2019; Reardon et al., 2019; Rizzo et al., 2023).

Similarly to other risk behaviours, social influences such as friends’ gambling and friends’ approval toward gambling were found to increase the probability of gambling behaviour (Canale et al., 2017; Hanss et al., 2014; Hurt et al., 2008; Lussier et al., 2014; Parrado-González et al., 2023; Riley et al., 2021). Finally, school factors such as truancy at school, school difficulties, and low grades were also found to be associated with adolescent gambling (Anagnostopoulos et al., 2017; Dickson et al., 2008; Molinaro et al., 2018). Indeed, lower-than-average academic performance, as measured by students’ self-reported grades in various subjects, including mathematics, was associated with gambling and risky gambling (Wahlström & Olsson, 2023). Maths grades and attitudes can be relevant since erroneous knowledge and understanding of mathematical/probabilistic concepts are recognized to cause gambling-related cognitive distortions influencing gambling behaviour (Delfabbro et al., 2009b; Donati et al., 2018; Raylu & Oei, 2004).

As described, previous studies identified a range of psychological, social, and environmental factors associated with gambling among adolescents. However, most studies were focused on problematic gambling behaviour and conducted on samples of adolescents older than 14 years of age. Given the multifaceted nature of gambling behaviour, the present study adopts an exploratory approach to examine a variety of correlated factors and broad patterns of gambling. The final aim is to comprehensively assess potential influences and generate insights for future hypothesis-driven research, as well as to provide information and knowledge suitable for designing effective school-based programmes to prevent gambling among early adolescents.

## 2. Materials and Methods

### 2.1. Study Design and Sample

This cross-sectional study used data from the baseline survey of the experimental controlled trial “GAPUnplugged” designed to evaluate the effectiveness of the GAPUnplugged school curriculum in preventing gambling among 12–14 years old adolescents. Study design and methods of the trial are described elsewhere (Vigna-Taglianti et al., 2024).

The survey involved 1874 students of 29 secondary schools located in the territories of nine National Health Service (NHS) districts (Rome, Alessandria, Torino 3, Torino 5, Vercelli, Cuneo 1, Cuneo 2, city of Torino, Novara) of Piedmont and Lazio Regions in Italy between November 2022 and January 2023. The analytical sample of the present study included 1822 students who answered the question on gambling behaviour in the last 30 days.

### 2.2. Data Collection and Data Management

A self-completed anonymous questionnaire was used to collect information on sociodemographic characteristics, substance use, gambling behaviours, beliefs, attitudes, risk perceptions and refusal skills towards gambling, perception of peers’ and friends’ substance use and gambling, friend’s approval of gambling, parental gambling, monitoring, support, permissiveness, school climate, attitudes toward maths and maths grades, self-esteem, impulsiveness, sensation-seeking, and antisocial behaviours.

The questionnaire was developed ad hoc and contained previously validated questions derived from the Unplugged evaluation survey “www.eudap.eu (accessed on 7 September 2022)”, the EDDRA data bank of EMCDDA “www.euda.europa.eu (accessed on 7 September 2022)”, and other international sources and projects (ESPAD, HBSC, Project ALERT, RATING Swedish cohort, SOGS-RA, BSSS). To preserve the confidentiality of the data, a 9-digit individual code was self-generated by the student before filling out the questionnaire.

Only students whose parents or caregivers gave consent to participate were involved in the study. Before the administration of the questionnaire, information on the study was provided to the pupils, and consent to participate was requested. The questionnaires were filled in by students in the classroom during school time through an online application. In cases of a lack of computers or problems of connection, the paper version of the questionnaire was administered.

### 2.3. Measures

Individual socio-demographic information included grade, age (based on birth date), gender, and languages spoken in the family.

Gambling behaviour was investigated by asking students if they gambled (scratch cards, lottery, bingo, slot machines, sport betting, event betting, poker, cards) during the last 30 days, with response categories ranging on a scale from 0 to 13 times or more for each specific game. A unique variable of gambling behaviour was created, and all the answers were summed up into a dichotomous indicator, “Yes” and “No”.

As regards psychological factors, attitudes, beliefs, risk perceptions, refusal skills, negative mood, impulsiveness, and sensation-seeking were investigated. Positive attitudes toward gambling were assessed through a 3-item scale: “I find it fun”, “I find it enthusiastic”, and “I will become rich”, ranking the answers on a 4-point Likert scale “Strongly agree”, “Agree”, “Disagree”, and “Strongly disagree”. The reliability of the scale was good (Cronbach’s alpha α = 0.68). Performance beliefs toward gambling were assessed through the following statements “I have an ability to predict my gambling winnings”, “Gambling is a sure way of becoming rich”, “If I gamble often, I have higher probability of winning”, “Winning and losing in gambling depends only on chance”, “Those who play sport have higher probability of winning the sports betting”, “If I come close to winning now, next time I will win”, and “In the lottery, if one number doesn’t come out for a long time, it will certainly come out soon”, ranking the answers on a 4-point Likert scale “Strongly agree”, “Agree”, “Disagree” and “Strongly disagree”. The reliability of the scale was good (Cronbach’s alpha α = 0.78). Risk perceptions were measured by using the question “How much do you think people risk harming themselves if they gamble” with possible answers “No risk”, “Slight risk”, “Great risk”, and “Don’t know”. Refusal skills were assessed through a situation question investigating students’ ability to cope with gambling offers, providing the following answers on a 4-point Likert scale: “Very likely”, “Likely”, “Unlikely”, and “Very unlikely”. The reliability of the Cronbach’s alpha was α = 0.60. Negative mood was measured by the two statements “I feel I have nothing to be proud of” and “I think I am a failure” of the Rosenberg Self-Esteem Scale (Rosenberg, 1965), allowing the response alternatives on a 4-point Likert scale “Strongly agree”, “Agree”, “Disagree”, and “Strongly disagree”. The reliability of the scale was good (Cronbach’s alpha α = 0.65). A 5-item scale investigated impulsiveness by asking for opinions on these statements: “I often say or do things without thinking”, “I often get in troubles because I do things without thinking it through”, “I am impulsive person”, “I weight up all the choices before I decide on something” (inversed), and “I often say something off the top of my head”, responding to a 4-point Likert scale with “Strongly agree”, “Agree”, “Disagree”, and “Strongly disagree”. The reliability of the scale was good (Cronbach’s alpha α = 0.77). Sensation-seeking was evaluated with the Italian version of Brief Sensation Seeking Scale (BSSS), including questions on experience seeking (e.g., “I would like to explore strange places”), boredom susceptibility (e.g., “I get restless when I spend too much time at home”), thrill and adventure seeking (e.g., “I like to do frightening things”), and disinhibition (e.g., “I like wild parties”), recording the responses on a 4-point Likert scale with “Strongly agree”, “Agree”, “Disagree”, and “Strongly disagree” (Primi et al., 2011). The reliability of the scale was good (Cronbach’s alpha α = 0.76). For all these variables, the answers of the Likert scales were scored from 1 to 4 and summed, means were calculated, and categories of high, middle, and low level of the indicator were created by using tertiles.

Questions on the perceived number of friends gambling allowed the answers: “None”, “Less than half of them”, “About half of them”, “More than half of them”, and “All of them”. The question regarding their friends’ approval of gambling allowed the answers “Would approve”, “Would disapprove but still be my friends”, “Would disapprove and stop being my friends”, and “They would not care”. The behaviour of gambling with friends was measured by asking “Have you ever gambled together with friends?” with possible answers including “Never gambled in general”, “Never gambled with friend”, “Sometimes”, and “Often”.

In terms of school behaviours, mathematics grades, attitudes toward mathematics, school performance, and respect for teachers were studied. Mathematics grades were investigated by a specific question, “My math grades are …”, allowing the answers “High”, “Medium”, and “Low”. A 4-item scale assessed the negative attitudes of the student toward mathematics (e.g., “I don’t like it”, “I don’t understand it”, “I make mistakes doing calculations”, “I don’t understand the formulas I have to apply”) allowing answers on a 4-point Likert scale such as “Strongly agree”, “Agree”, “Disagree”, and “Strongly disagree”. The reliability of the scale was good (Cronbach’s alpha α = 0.82). These answers were scored 1 to 4 and summed, means were calculated, and categories of high, middle, and low level were created by using tertiles. School performance and pupil’s respect for teachers were assessed by a single-item scale “How I do in school matters a lot to me” and “I have great respect for what my teachers tell me”, respectively, allowing answers on a 4-point Likert scale such as “Strongly agree”, “Agree”, “Disagree”, and “Strongly disagree”. Further details on the measures are provided in the study design paper (Vigna-Taglianti et al., 2024).

### 2.4. Statistical Analysis

The outcome under study was gambling in the last 30 days (yes/no).

Descriptive statistics were summarized through frequency and percentage for categorical variables and mean and SD for continuous variables.

The associations of sociodemographic characteristics, attitudes, and beliefs toward gambling, risk perceptions, refusal skills, negative mood, impulsiveness, sensation-seeking, normative perceptions of friend’s gambling, friends’ approval of gambling, gambling with friends, mathematics grades, negative attitudes toward mathematics, school performance, and respect for teachers with the probability of adolescent’s gambling in the last 30 days were estimated through bivariate regression models. Collinearity between variables was checked before building the final model. Non-collinear and statistically significant variables from the bivariate model were included in the final multivariate regression model simultaneously. Some variables were collinear: “Age” and “Grade” (r = 0.8); “Negative attitudes toward mathematics” and “Mathematics grades” (r = 0.6). We included in the multivariate model the variables “Age” and “Mathematics grades” due to their stronger significance in the bivariate model.

Multilevel mixed-effect modelling was used to control for the hierarchical nature of the data, with two grouping levels: centre as I level, and class as II level. The LR test showed that adding the third level “school” did not make a statistically significant difference, so the two-level model was used. The variable “Centre” was recategorized, merging NHS districts with low sample according to context similarities, so the final variable had four levels, i.e., Rome, Torino3/Torino5/Torino, Vercelli/Cuneo1/Cuneo2, and Alessandria/Novara. Adjusted Odds Ratios (AORs) and 95% Confidence Interval (95%CI) were estimated as measures of association between the studied factors and the outcome. Some categorical variables were re-coded in order to reduce the number of items included in the model, i.e., categories were merged. Missing data were less than 6% for all studied variables. Applying listwise deletion to handle missing data, the final model was run on 1565 students (86% of the initial sample).

Statistical analysis was carried out using STATA software release 18.0 (Stata Corporation, 2023).

## 3. Results

### 3.1. Descriptive Statistics

**Figure 1:**
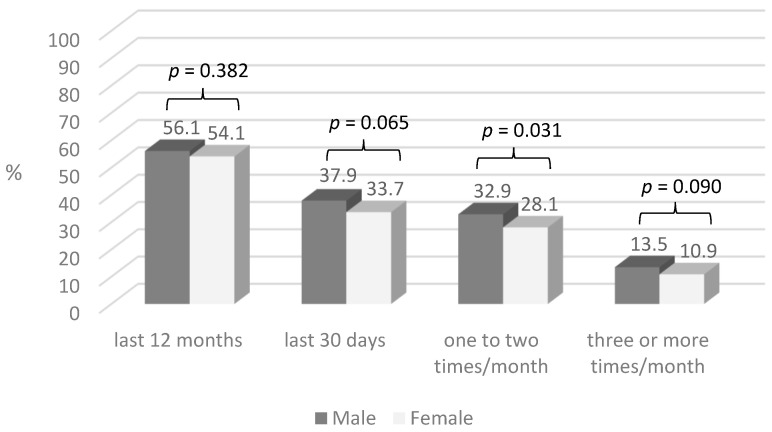
Prevalence of gambling behaviours by gender.

The overall prevalence of last 30 days gambling behaviour was 36.4%, of which 37.9% among males and 33.7% among females (*p* = 0.065) (Figure 1). A significantly higher proportion of males than females gambled one to two times a month (32.9% vs. 28.1%, *p* = 0.031).

Socio-demographic and school behaviours of the pupils are described in Table 1. The mean age was 13.1 (SD ± 0.8). Age did not differ significantly by gambling group. Third-grade students of the first cycle were significantly more prevalent in the gambling vs. non-gambling group (*p* = 0.004). Regarding the languages spoken in the family, the proportion of students from non-Italian speaking families was higher in the gambling group (28.6% vs. 24.0%, *p* = 0.029). Only Arabic- and Chinese/Indian/Philippines-speaking families were more prevalent in the non-gambling group. Low mathematic grades (21.6% vs. 17.6%, *p* = 0.019), low school performance (17.9% vs. 11.2%, *p* < 0.001), and low respect for teachers (15.2% vs. 9.8%, *p* = 0.001) were more prevalent in the gambling group. Attitudes toward mathematics did not differ between groups.

In terms of psychological factors, high positive attitudes (43.7% vs. 19.3%, *p* < 0.001), high performance beliefs toward gambling (40.4% vs. 20.5%, *p* < 0.001), low and slight risk perceptions (37.4% vs. 21.0% and 29.6% vs. 25.1%, *p* < 0.001), and low refusal skills (36.1% vs. 16.8%, *p* < 0.001) were significantly higher among pupils who gambled in the last 30 days. Other characteristics such as having high levels of negative mood (23.2% vs. 18.2%, *p* = 0.021), high impulsiveness (39.0% vs. 28.4%, *p* < 0.001), and high sensation-seeking (38.3% vs. 27.3%, *p* < 0.001) were also significantly higher in the gambling group (Table 2).

As regards norms, perception of friends gambling (43.6% vs. 21.0%, *p* < 0.001) was significantly higher in the gambling group. A higher proportion of pupils who gambled reported their friends would approve of gambling compared to non-gamblers (54.6% vs. 30.0%, *p* < 0.001). Gambling with friends sometimes and often (20.2% vs. 5.5% and 5.1% vs. 0.8%, *p* < 0.001) was significantly higher among pupils who gambled (Table 2).

### 3.2. Multilevel Regression Model

In the multivariate multilevel regression model, some factors (languages in family, school behaviours, refusal skills, negative mood, impulsiveness, and sensation seeking) lost significance.

High positive attitudes toward gambling (OR 1.72, 95%CI 1.26–2.34) were confirmed as independent correlate of adolescent’s gambling behaviour. High performance beliefs toward gambling were associated with 67% higher probability of recent gambling. Slight and low risk perceptions toward gambling were associated with 68% to 98% higher probability of gambling, respectively.

Adolescents who perceived their friends gambled were 1.71 times more likely to engage in gambling (OR 1.71, 95%CI 1.31–2.23). The odds of adolescent’s last 30 days gambling was highest if their friends approved gambling (OR 2.92, 95%CI 1.95–4.37) and would not care if they gambled (OR 1.88, 95%CI 1.28–2.78), as well as if they gambled with friends (OR 2.78, 95%CI 1.90–4.06) (Table 3).

## 4. Discussion

The present study investigated the association of psychological factors, school behaviours, and normative perceptions with the probability of recent gambling among 1822 secondary school students in Italy. The prevalence of gambling behaviour was very high, with 55.7% of participants (12–14 years old) declaring they have gambled at least once in the last year and 36.4% at least once in the last month. Results showed that attitudes toward gambling, performance beliefs, risk perceptions, and normative perceptions were independent significant correlates of adolescents’ recent gambling behaviour.

The prevalence of last year’s gambling was similar to the 52% prevalence found among 15-year-old students in the study by Favieri et al. (2023), but higher than the 32% found among 16-year-old Italian ESPAD students (Molinaro et al., 2020). However, the sample of students participating in our study were younger than 14 years of age, confirming that gambling behaviour is a worrisome problem even in early ages, although we cannot compare our results with other surveys conducted on early adolescents. Given the precocious age of the study participants, it appears essential to implement prevention programmes focused on gambling behaviour in lower secondary schools and other prevention strategies in the larger community to reduce this risky behaviour.

Adolescents with high positive attitudes toward gambling, i.e., those who stated that they considered gambling fun, exciting, and a chance to become rich, had about 70% higher probability of involvement in gambling behaviour. The role of beliefs and attitudes as determinants of gambling behaviour is well recognized (Derevensky & Gilbeau, 2015; Griffiths & Wood, 2000; Hanss et al., 2014; Hurt et al., 2008; Pallesen et al., 2016). Attitudes and beliefs form from the influence of the general environment, including society, the family, and the media. The latter have a strong role in gambling attitudes of adolescents, e.g., a positive image of gambling is shared by social media that are widely used by adolescents (Delfabbro et al., 2016; O’Loughlin & Blaszczynski, 2018). As a consequence, gambling represents a fun, exciting, and get-rich-quick activity for adolescents (Hurt et al., 2008; Vegni et al., 2019).

High performance beliefs toward gambling, i.e., the perception of knowing gambling rules and how to win, and slight or low risk perceptions toward gambling were significantly correlated with gambling behaviour. Similarly, Favieri et al. (2023) found that 15-year-old adolescents who gamble have greater gambling-related expectancies, illusion of control, predictive control, perceived inability to stop gambling, and interpretative control. This illusion of control may lead adolescents to believe they can control the negative outcomes of the gaming experience (Favieri et al., 2023). Other studies have shown that adolescent gambling is associated with incorrect point of view on the randomness, superstitious beliefs, optimistic attitudes towards the profitability of gambling, and poor perception of the risks associated with gambling (Botella-Guijarro et al., 2020; Delfabbro et al., 2006; Delfabbro et al., 2009a; Delfabbro & Thrupp, 2003; Donati et al., 2013). Adolescents who gamble may not perceive the random nature of gambling but rather have a distorted vision about the real risk of losing money, the impossibility of controlling the game itself, and the prediction of winnings, as they consider gambling to be a harmless and risk-free activity. Furthermore, these aspects are corroborated by the great release of adrenaline that occurs during gambling, which determines a disconnection from reality (Bullock & Potenza, 2012).

Our study confirms the important role of normative beliefs in influencing risk behaviours. Indeed, friends’ gambling, friends’ approval of gambling, and gambling with friends were significantly correlated with adolescent gambling behaviour (Hanss et al., 2014; Hurt et al., 2008; Parrado-González et al., 2023; Riley et al., 2021). A behaviour perceived as being adopted by one’s friends is considered needed to be accepted into the group and be part of it. Behaviours shared and appreciated by friends and perceived as acted by the majority of peers may be considered as correct and risk-free. This concept is in line with the social learning theory, which suggests that behaviours are learned through imitation, modelling, and observation of social interactions with others (Bandura, 1986; Bandura & Walters, 1977).

Finally, we cannot confirm the role of negative mood, impulsiveness, and sensation seeking as independent correlates of gambling behaviour among 12–14-year-old adolescents. These factors indeed lost significance in the adjusted model. The prevalence was not low; therefore, the inconsistency is more likely due to differences in the sample of students analyzed by the studies. Also, it is arguable that other factors are more important in influencing the gambling behaviour in this age group.

This study has several strengths. The surveys used standardized questionnaires containing previously validated questions derived from recognized international sources, minimizing possible misclassifications related to data collection and measures. Multilevel mixed-effect regression models were performed to evaluate the association between correlates and gambling, according to higher-order clustering (centre and class). The information collected in the survey allowed the analysis of a large set of correlates. However, this study should be considered in light of some limitations. The cross-sectional nature of the study prevents inferring causality. Missing values reduced the sample in the adjusted regression models; however, the models were run on 86% of the sample, which is a large sample. All the information was self-reported by the students; therefore, the students’ perceptions of their friends’ gambling and friends’ approval of gambling may not accurately reflect the actual behaviour. Nevertheless, we consider the students’ perceptions of the behaviour and approval of their friends to be important because it may have an even stronger impact on their behaviour. Furthermore, this is a secondary analysis of the study, i.e., the study was designed to evaluate the impact of the school prevention curriculum on reducing gambling behaviour, and this could limit the representativeness of this study. The sample was not equally distributed between the nine centres; therefore, we needed to merge some centres according to similarity between them to perform multilevel analysis by centre.

The results of the study highlight the need to design large and coordinated preventive strategies to prevent gambling in adolescence. In this light, limiting the availability and accessibility of gambling opportunities appears to be of paramount importance. Schools should educate to raise awareness of the harms and the impact of gambling on relationships, finances, and mental health (Giménez Lozano & Morales Rodríguez, 2022; Monreal-Bartolomé et al., 2023). In this regard, promoting the comprehension of complex mathematical concepts such as randomness and expected value can help adolescents to understand the unprofitability and unpredictability of gambling. Indeed, it has been found that focusing only on the negative aspects of gambling and its consequences is not sufficient to prevent gambling behaviour (Keen et al., 2017). Finally, the interventions should use new technologies and multimedia elements to be more attractive to adolescents (Monreal-Bartolomé et al., 2023; St-Pierre & Derevensky, 2016).

In conclusion, the present study identified some psychological factors, school behaviours, and normative perceptions as independent correlates of gambling among adolescents: attitudes toward gambling, performance beliefs, risk perceptions toward gambling, friends’ gambling, friends’ approval of gambling, and gambling with friends. These factors should be taken into account to design and implement preventive strategies to reduce gambling behaviours among early adolescents and limit health consequences in later ages.

## Figures and Tables

**Table 1:** Socio-demographic and school behaviours of pupils who gamble vs. those who do not gamble in the last 30 days.

Characteristics	Overall (N = 1822)		Gambling (N = 663)		No Gambling (N = 1159)		*p*-Value
	N	%	N	%	N	%	
Grade (secondary schools)							
II grade first cycle	977	53.6	360	54.3	617	53.2	0.004
III grade first cycle	693	38.0	266	40.1	427	36.8	
I grade second cycle	152	8.3	37	5.6	115	9.9	
Age							
12	515	28.3	179	27.0	336	29.0	0.472
13	813	44.6	308	46.5	505	43.6	
14	494	27.1	176	26.6	318	27.4	
Mean ± SD	13.1 ± 0.8		13.1 ± 0.8		13.0 ± 0.8		0.472
Gender							
Female	829	48.1	279	45.2	550	49.8	0.065
Male	894	51.9	339	54.8	555	50.2	
Languages in the family							
Italian	1349	74.3	471	71.4	878	76.0	0.029
Others	466	25.7	189	28.6	277	24.0	
Languages in the family							
Italian/English/German/French	1386	76.1	488	73.6	898	77.5	0.022
Spanish/Portuguese	71	3.9	30	4.5	41	3.5	
Arabic	76	4.2	26	3.9	50	4.3	
Slavic/Russian/Albanian	76	4.2	28	4.2	48	4.1	
Chinese/Indian/Philippines	41	2.3	10	1.5	31	2.7	
Other	172	9.4	81	12.2	91	7.9	
Mathematics grades							
High	584	32.5	188	28.8	396	34.5	0.019
Middle	872	48.5	323	49.5	549	47.9	
Low	343	19.1	141	21.6	202	17.6	
Negative attitudes toward mathematics							
Low	707	39.1	236	36.0	471	40.8	0.086
Middle	614	33.9	226	34.5	388	33.6	
High	488	27.0	193	29.5	295	25.6	
School performance							
High	1562	86.4	538	82.1	1024	88.8	<0.001
Low	246	13.6	117	17.9	129	11.2	
Respect for teachers							
High	1590	88.2	553	84.8	1037	90.2	0.001
Low	212	11.8	99	15.2	113	9.8	

SD—Standard deviation.

**Table 2:** Attitudes, beliefs, risk perceptions, skills, mood, impulsiveness, sensation seeking, and normative perceptions of pupils gambling vs. no gambling in the last 30 days.

Characteristics	Overall (N = 1822)		Gambling (N = 663)		No Gambling (N = 1159)		*p*-Value
	N	%	N	%	N	%	
Positive attitudes toward gambling							
Low	926	51.4	242	36.9	684	59.8	<0.001
Middle	366	20.3	127	19.4	239	20.9	
High	508	28.2	287	43.7	221	19.3	
Performance beliefs toward gambling							
Low	719	40.1	167	25.6	552	48.5	<0.001
Middle	576	32.1	222	34.0	354	31.1	
High	497	27.7	264	40.4	233	20.5	
Risk perceptions toward gambling							
High risk perceptions	522	28.7	121	18.2	401	34.6	<0.001
Slight risk perceptions	487	26.7	196	29.6	291	25.1	
Low risk perceptions	491	27.0	248	37.4	243	21.0	
Don’t know/miss	322	17.7	98	14.8	224	19.3	
Refusal skills on gambling							
High	680	37.5	165	25.0	515	44.6	<0.001
Middle	701	38.7	256	38.9	445	38.6	
Low	432	23.8	238	36.1	194	16.8	
Negative mood							
Low	1041	58.9	372	57.9	669	59.4	0.021
Middle	373	21.1	121	18.9	252	22.4	
High	354	20.0	149	23.2	205	18.2	
Impulsiveness							
Low	754	41.9	223	34.2	531	46.2	<0.001
Middle	466	25.9	175	26.8	291	25.4	
High	581	32.3	255	39.0	326	28.4	
Sensation seeking							
Low	741	41.4	217	33.5	524	45.9	<0.001
Middle	488	27.3	183	28.2	305	26.7	
High	560	31.3	248	38.3	312	27.3	
Perception of friends’ gambling							
No	1276	70.8	370	56.4	906	79.0	<0.001
Yes	527	29.2	286	43.6	241	21.0	
Friends’ approval of gambling							
Would not approve	342	19.0	53	8.0	289	25.3	<0.001
Would approve	702	39.0	359	54.6	343	30.0	
Would not care	757	42.0	246	37.4	511	44.7	
Gambling with friends							
Never in general/with my friends	1567	86.9	487	74.7	1080	93.7	<0.001
Sometimes	195	10.8	132	20.2	63	5.5	
Often	42	2.3	33	5.1	9	0.8	

**Table 3:** Factors associated with gambling in the last 30 days.

Correlates	COR (95% CI)	*p*-Value	AOR (95% CI)	*p*-Value
	N = 1822		N = 1565	
Grade (ref: second grade, middle school)				
Third grade middle school	1.19 (0.89–1.59)	0.228	-	
First grade high school	0.62 (0.32–1.20)	0.155		
Age (continuous)	1.11 (0.93–1.33)	0.236	0.95 (0.74–1.24)	0.726
Gender (ref: female)				
Male	1.20 (0.97–1.47)	0.091	1.07 (0.84–1.38)	0.576
Languages in the family (ref: Italian)				
Others	1.27 (1.01–1.60)	**0.038**	1.21 (0.91–1.62)	0.191
Mathematics grades (ref: high)				
Middle	1.27 (1.01–1.61)	**0.039**	1.15 (0.87–1.53)	0.324
Low	1.60 (1.20–2.15)	**0.002**	1.33 (0.92–1.93)	0.127
Negative attitudes toward mathematics (ref: low)				
Middle	1.16 (0.92–1.47)	0.212	-	
High	1.38 (1.08–1.78)	**0.011**		
School performance (ref: high)				
Low	1.80 (1.36–2.39)	**<0.001**	1.07 (0.74–1.55)	0.720
Respect for teacher (ref: high)				
Low	1.64 (1.21–2.22)	**0.001**	0.99 (0.66–1.50)	0.993
Positive attitudes toward gambling (ref: low)				
Middle	1.49 (1.14–1.96)	**0.003**	1.05 (0.76–1.45)	0.755
High	3.67 (2.89–4.65)	**<0.001**	1.72 (1.26–2.34)	**0.001**
Performance beliefs toward gambling (ref: low)				
Middle	2.02 (1.58–2.59)	**<0.001**	1.29 (0.95–1.73)	0.098
High	3.68 (2.85–4.75)	**<0.001**	1.67 (1.20–2.33)	**0.003**
Risk perceptions toward gambling (ref: high risk perceptions)				
Slight risk perceptions	2.22 (1.68–2.94)	**<0.001**	1.68 (1.21–2.33)	**0.002**
Low risk perceptions	3.28 (2.48–4.33)	**<0.001**	1.98 (1.41–2.79)	**<0.001**
Don’t know/miss	1.42 (1.03–1.96)	**0.033**	1.06 (0.72–1.56)	0.773
Refusal skills on gambling (ref: high)				
Middle	1.81 (1.43–2.30)	**<0.001**	1.15 (0.86–1.55)	0.355
Low	3.91 (2.99–5.11)	**<0.001**	1.28 (0.89–1.84)	0.181
Negative mood (ref: low)				
Middle	0.85 (0.66–1.11)	0.233	0.82 (0.60–1.12)	0.211
High	1.28 (0.99–1.66)	0.056	0.84 (0.61–1.16)	0.289
Impulsiveness (ref: low)				
Middle	1.43 (1.11–1.84)	**0.006**	0.97 (0.71–1.32)	0.830
High	1.82 (1.44–2.31)	**<0.001**	1.10 (0.80–1.49)	0.559
Sensation seeking (ref: low)				
Middle	1.44 (1.12–1.85)	**0.005**	0.88 (0.65–1.20)	0.420
High	1.91 (1.51–2.43)	**<0.001**	0.97 (0.71–1.32)	0.829
Perception of friends’ gambling (ref: no)				
Yes	2.86 (2.30–3.55)	**<0.001**	1.71 (1.31–2.23)	**<0.001**
Friends’ approval of gambling (ref: would not approve)				
Would approve	5.68 (4.06–7.95)	**<0.001**	2.92 (1.95–4.37)	**<0.001**
Would not care	2.59 (1.85–3.62)	**<0.001**	1.88 (1.28–2.78)	**0.001**
Gambling with friends (ref: never in general/with my friends)				
Sometimes/Often	4.99 (1.67–6.79)	**<0.001**	2.78 (1.90–4.06)	**<0.001**

Multilevel mixed-effect models controlled for two levels: centre and class. Crude Odds Ratios (COR); Adjusted Odds Ratios (AOR); 95% Confidence Interval (95%CI). Statistically significant results are marked in bold.

## Data Availability

Data will be provided upon request. Federica Vigna-Taglianti is responsible for the data.
